# Cost-Effectiveness of Rheumatic Heart Disease Echocardiographic Screening in Brazil: Data from the PROVAR+ Study: Cost-effectiveness of RHD screening in Brazil

**DOI:** 10.5334/gh.529

**Published:** 2020-02-20

**Authors:** Jasper Ubels, Craig Sable, Andrea Z. Beaton, Maria Carmo P. Nunes, Kaciane K. B. Oliveira, Lara C. Rabelo, Isabella M. Teixeira, Gabriela Z. L. Ruiz, Letícia Maria M. Rabelo, Alison R. Tompsett, Antonio Luiz P. Ribeiro, Klas-Göran Sahlen, Bruno R. Nascimento, On behalf of the PROVAR+ (Programa de RastreamentO da VAlvopatia Reumática e Outras Doenças Cardiovasculares) investigators

**Affiliations:** 1 Department of Epidemiology and Global health, Umeå University, Umeå, Västerbotten, SE; 2 Children’s National Health System, Washington, DC, US; 3 The Heart Institute, Cincinnati Childrens Hospital Medical Center, Cincinnati, OH, US; 4 Serviço de Cardiologia e Cirurgia Cardiovascular e Centro de Telessaúde, Hospital das Clínicas da Universidade Federal de Minas Gerais, Belo Horizonte, MG, BR; 5 Departamento de Clínica Médica, Faculdade de Medicina da Universidade Federal de Minas Gerais, Belo Horizonte, MG, BR; 6 Edwards Lifesciences Foundation, US

**Keywords:** rheumatic heart disease, echocardiography, screening, cost-effectiveness

## Abstract

**Introduction:**

In recent years, new technologies – noticeably ultra-portable echocardiographic machines – have emerged, allowing for Rheumatic Heart Disease (RHD) early diagnosis. We aimed to perform a cost-utility analysis to assess the cost-effectiveness of RHD screening with handheld devices in the Brazilian context.

**Methods:**

A Markov model was created to assess the cost-effectiveness of one-time screening for RHD in a hypothetical cohort of 11-year-old socioeconomically disadvantaged children, comparing the intervention to standard care using a public perspective and a 30-year time horizon. The model consisted of 13 states: No RHD, Undiagnosed Asymptomatic Borderline RHD, Diagnosed Asymptomatic Borderline RHD, Untreated Asymptomatic Definite RHD, Treated Asymptomatic Definite RHD, Untreated Mild Clinical RHD, Treated Mild Clinical RHD, Untreated Severe Clinical RHD, Treated Severe Clinical RHD, Surgery, Post-Surgery and Death. The initial distribution of the population over the different states was derived from primary echo screening data. Costs of the different states were derived from the Brazilian public health system database. Transition probabilities and utilities were derived from published studies. A discount rate of 3%/year was used. A cost-effectiveness threshold of $25,949.85 per Disability Adjusted Life Year (DALY) averted is used in concordance with the 3x GDP per capita threshold in 2015.

**Results:**

RHD echo screening is cost-effective with an Incremental Cost-Effectiveness Ratio of $10,148.38 per DALY averted. Probabilistic modelling shows that the intervention could be considered cost-effective in 70% of the iterations.

**Conclusion:**

Screening for RHD with hand held echocardiographic machines in 11-year-old children in the target population is cost-effective in the Brazilian context.

**Highlights:**

- A cost-effectiveness analysis showed that Rheumatic Heart Disease (RHD) echocardiographic screening utilizing handheld devices, performed by non-physicians with remote interpretation by telemedicine is cost-effective in a 30-year time horizon in Brazil.
- The model included primary data from the first large-scale RHD screening program in Brazilian underserved populations and costs from the Unified Health System (SUS), and suggests that the Incremental Cost-Effectiveness Ratio of the intervention is considerably below the acceptable threshold for Brazil, even after a detailed sensitivity analysis.
- Considering the high prevalence of subclinical RHD in Brazil, and the significant economic burden posed by advanced disease, these data are important for the formulation of public policies and surveillance approaches.
- Cost-saving strategies first implemented in Brazil by the PROVAR study, such as task-shifting to non-physicians, computer-based training, routine use of affordable devices and telemedicine for remote diagnosis may help planning RHD control programs in endemic areas worldwide.

## Introduction

Rheumatic Heart Disease (RHD) is a significant worldwide problem, with an estimated global prevalence of nearly 33 million people in 2015 [1], resulting in at least 310,000 premature deaths every year [1]. Important identified societal risk factors for RHD are poverty, malnutrition, underemployment, maternal education and overcrowding [2]. As children and adolescents are particularly affected [3,4,5], the disease adds an extra burden on an already disadvantaged population.

RHD late sequelae – markedly heart failure – have big impacts on healthcare budgets of endemic countries, with increasing costs as disease advances, especially for treatment of heart failure, which often involves heart surgery and other expensive interventions. Thus, preventive measures intervening in the course of RHD might thus not only be medically effective, but also economically advantageous. Secondary prophylaxis with penicillin is associated with a 50–70% regression of clinical RHD [6] and, in Brazil, Benzathine Penicillin G (BPG) is administered every 3 to 4 weeks [7]. The length of secondary prophylaxis depends on the severity of RHD and is in some cases recommended for life.

The Sistema Único de Saúde (SUS), the Brazilian public health system, reported a cost of 33 million USD for 14,010 hospitalizations associated with acute RHD in 2013 [3]. The first large-scale screening program conducted in the country [3] found a high prevalence of asymptomatic RHD (4.2%) as compared to data from other underserved countries. New in this study was the use of handheld echocardiographic machines, operated by non-physicians. These devices are inexpensive and easy to operate, making it possible to conduct screening programs in non-hospital settings such as schools and community health centers. Published data suggests early detection through screening might also be cost-effective or even cost-saving from a public and societal point of view, if the intervention prevents progression to clinical RHD [4,8,9]. However, to date no cost-effectiveness analysis has been performed in South America.

The aim of this study is to assess the cost-effectiveness of a targeted screening program for RHD early detection with handheld echo machines in Brazil.

## Methods

The “*Programa de RastreamentO da Valvopatia Reumática*” (PROVAR) is the RHD echocardiographic screening program conducted in Belo Horizonte and two other smaller cities in the north of the state of Minas Gerais, Southeast Brazil, since 2014. Detailed information about the screening intervention has been published elsewhere [3]. The current study analyses a hypothetical screened cohort based on data from the PROVAR study. Specifically, the targeted population is economically disadvantaged 11-year-old children in the state of Minas Gerais, without prior known history of Acute Rheumatic Fever (ARF) or RHD. A hypothetical cohort of 1,000 enrolled children is followed until the age of 41 (30 cycles). The choice of the age of 11 was made based on prevalence data from Nascimento et al. [3,10]. Identifying and preventing RHD progression at an early stage would theoretically yield the highest effect [8]. The decision to run the model for 30 cycles until the hypothetical age of 41 instead of a full life cycle was based on the fact that little is known about how RHD behaves in adult populations compared to the knowledge about the course of RHD in childhood.

### Model

A Markov model was used to evaluate the effect of RHD echocardiographic screening compared to standard care. A Markov model is a model that tries to, in this case, simulate the behaviour of a disease in a population and how an intervention influences that behaviour [11]. The model is built with different mutually exclusive states that together model the course of disease. Movements between different states are based on the movement of proportions of one state to another, the so-called transition probabilities. These movements happen per cycle; in this study, each cycle represents one year. Individual states are assigned their own utility weight, in this analysis Disability Adjusted Life Years (DALYs), and associated costs related to the specific state. Two cohorts were modelled: one under standard care and one with a screening program.

Under current practice only a fraction of the target population affected by RHD seek medical care, frequently presenting to health services when RHD becomes clinically symptomatic. The initial distribution of the population was derived from the PROVAR study [3,10]. Further information about the distribution of the population over the states can be found in the Appendix.

Utility and costs of the standard care cohort were compared with the utility and costs under intervention over a time horizon of 30 years. The outcome of the study is an Incremental Cost-Effectiveness Ratio (ICER). The predictions of the model were externally validated with prevalence data from the study by Nascimento et al. [3,10] by comparing the prevalence of Asymptomatic Borderline RHD and Asymptomatic Definite RHD in the age groups 11 to 13.9 and 14 to 18 predicted by the model with the respective observed prevalence in the respective age groups in the study [3] (results in Appendix).

**Figure 1:**
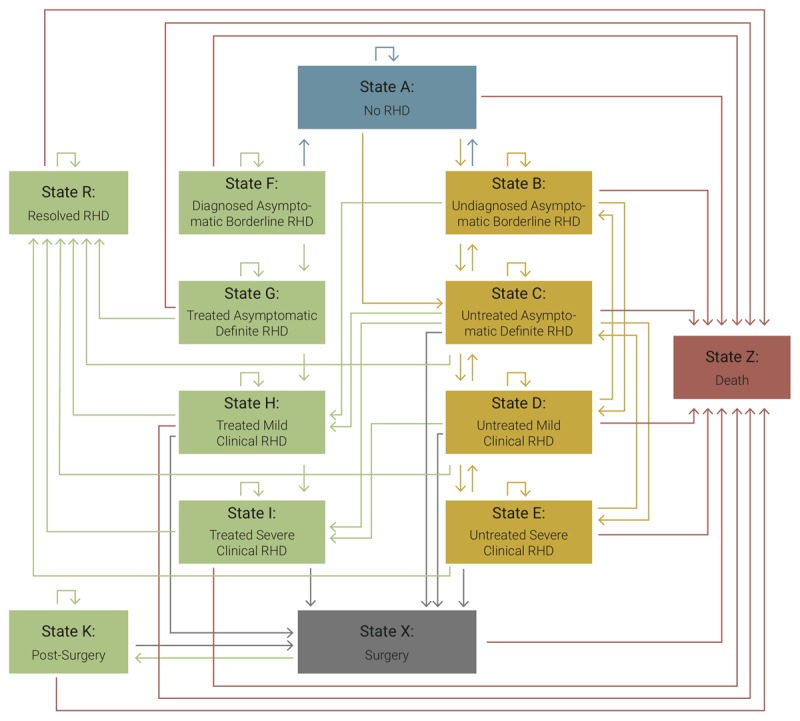
Graphical representation of the Markov model applied for the evaluation of systematic Rheumatic Heart Disease screening in Brazil.

A graphical representation of the model is shown in Figure 1. The effect of screening is assumed to be that because of early detection, a higher proportion of the cohort receives appropriate RHD care. The core of the disease course in this study is based on the different states, from (asymptomatic) borderline and definite RHD as defined by the World Heart Federation (WHF) [12] to stages of symptomatic disease based on the Australian RHD guidelines [13]. A detailed description of the model can be found in the Appendix. Initial distributions for the different states are derived from the study by Nascimento et al. [3].

### Costs

A public perspective was used to calculate the costs, which means that only direct costs were calculated. The costs are presented in 2017 United States Dollars (USD). Currency conversions were calculated with the website www.xe.com [14] (“XE Currency Converter – Live Rates,”) [15]. When necessary, costs were inflated to 2017 prices. In Brazil, a multitude of different inflation indices exist. In this study the decision was made to calculate the inflation of the Brazilian Reais (R$) with the index “*Índice Nacional de Preços ao Consumidor Amplo*” (IPCA-E) [16]. This inflation index corrects inflation for commodities used by Brazilians that earn 1 to 40 times the minimum wage in metropolitan areas such as Belo Horizonte.

Most of the costs estimates of RHD care for the public health system in Brazil are based on primary data from the SUS databases (Medical Procedure List [LPM 1999] reimbursement tables) and the PROVAR project; only the annual costs for post-surgery and severe clinical RHD patients were assumed. These costs were assumed to be similar to post-surgery costs of patients with ischemic heart disease, derived from a published study [17]. The costs consisted of consultations, medication, hospitalizations, medical exams, and procedures. Screening costs were estimated from the PROVAR project. It was calculated that the cost per scan was $6.60. Furthermore, due to false positives of screening, extra follow-up costs were also incurred.

Table 1 depicts the aggregated costs per state per individual. In the Appendix, a detailed overview of all the cost calculations is provided. A gamma distribution was used for the probabilistic analysis of the costs. Variance of all the parameters was assumed to be equal to the variance of state X: *Surgery*, which was the only state from which variance could be obtained. Information about the variance of costs of surgery was derived from the DATASUS, the informatics department of the health care system of Brazil.

**Table 1:** Costs per individual per state (in 2017 dollars).

State	Cost	Source

Stata A: No RHD	0	–
State B: Undiagnosed Asymptomatic Borderline RHD	0	–
State C: Untreated Asymptomatic RHD	0	–
State D: Untreated Mild Clinical RHD	0	–
State E: Untreated Severe Clinical RHD	0	–
State F: Diagnosed Borderline RHD	$25.84	DataSUS
State G: Treated Asymptomatic RHD	$93.93	DataSUS
Stage H: Treated Mild Clinical RHD	$337.47	DataSUS
State I: Treated Severe Clinical RHD	$854,00	Ribeiro et al. [17]
State RG: Resolved RHD	$93.93	Assumed to be equal to stage G
State RH: Resolved RHD	$337.47	Assumed to be equal to state H
State RI: Resolved RHD	$854,00	Assumed to be equal to state I
State K: Post Surgery	$854,00	Ribeiro et al. [17]
State X: Surgery	$4,120.51	dataSUS, based on disease codes from do Espirito Santo Freire et al. [33]
State Z: Death	0	–

**Abbreviations:** DataSUS: administrative database of the public health system (Unified Health System) in Brazil; RHD: Rheumatic Heart Disease.

### Outcome

DALY weights for the different RHD stages were derived from the Global Burden of Disease 2016 study [18]. Appendix Table 7 provides an overview of all the states with their respective utility weights in DALYs [19]. A similar approach was used in the study by Roberts et al. [8]. Furthermore, it was assumed that there is a disutility associated with knowing that an individual has Asymptomatic Borderline RHD [19]. In the probabilistic analysis a triangular distribution was used between the 95% uncertainty intervals provided by the Global Burden of Disease study 2016 [18]. To calculate the years of life lost (YYL), life expectancy by age of the modelled cohort was assumed to be similar to those living in the most socioeconomically disadvantaged areas of Rio de Janeiro [20].

### Discount rate

A discount rate of 3% for both costs and outcomes was used, as recommended by the World Health Organization [21].

### Transition probabilities

Figure 1 shows the possible transitions between states and Appendix Table 1 their respective probabilities. Transition probabilities were based on a mix of primary data, secondary data and assumptions. The Appendix gives detailed information about the sources of data and assumptions made for various transition probabilities.

The effect of secondary prophylaxis was assumed to be a reduction in RHD progression, similar to the assumptions by Roberts et al. [8]. It was assumed that adhering to secondary prophylaxis would result in a 50% reduction in disease progression from asymptomatic definite RHD to clinical RHD – with additional sensitivity analyses for the assumption of lower reduction effects – and a 50% reduction from mild clinical RHD to severe clinical RHD. These assumptions were based on the effects of BPG on the relapses of ARF [22].

The sensitivity of the effect of secondary prophylaxis on asymptomatic definite RHD was tested by reducing the preventive effect from 50% to no effect at all. The sensitivity of the effect of secondary prophylaxis on clinical RHD was tested by increasing the effect to a 75% reduction in disease progression and a 25% reduction in disease progression.

### Probabilistic analysis

Probabilistic analysis was conducted to control for variance in the transition probabilities. This was done by rerunning the Markov model for 10,000 iterations and then calculating the percentage of iterations either above or under a cost-effectiveness threshold. An overview of the type of distribution used per parameter is shown in Appendix Table 1. Yearly counts of the events were estimated by using yearly probabilities when these were missing from secondary data. When multiple different sources were used for describing the transition probabilities of one state it was decided to include the highest variance in the probabilistic analysis.

### One-way sensitivity analysis

One-way sensitivity analysis was performed on all the parameters. Transition probabilities and costs associated with the states increased and decreased according to the 95% confidence intervals’ upper – and lower boundaries of the respective parameters. DALY weights were increased and decreased with their respective 95% uncertainty intervals presented in the Global Burden of Disease 2016 study [18]. The sensitivity of the results to the discount rate was tested by varying the discount rate from 0% to 5%. The sensitivity of screening was tested by increasing the sensitivity to 100% and decreasing it to 80% in the case of Asymptomatic Definite RHD, and increasing the sensitivity to 80% and decreasing it to 50% in the case of Asymptomatic Borderline RHD. Further information about sensitivity analysis of medication adherence can be found in the Appendix and Appendix Table 8.

## Results

### Incremental costs and outcomes

Table 2 presents the results of the deterministic analysis. After running the hypothetical cohort for 30 cycles, the ICER was $10,148.38 per DALY averted and could be considered cost-effective under the implicit cost-effectiveness threshold of Brazil of $25,949.85, which is three times the GDP per capita in 2015 [23]. This implicit threshold is suggested by a draft report from the Brazilian Ministry of Health to be the maximum threshold [23,24].

**Table 2:** Results of the deterministic cost-effectiveness analysis.

Parameter	Cost

Cost standard treatment	$34,249.72
Cost intervention (screening)	$49,828.52
DALY’s standard treatment	726.63
DALY’s intervention	725.09

**Abbreviations:** DALY: Disability-adjusted life years.

### Probabilistic analysis

**Figure 2:**
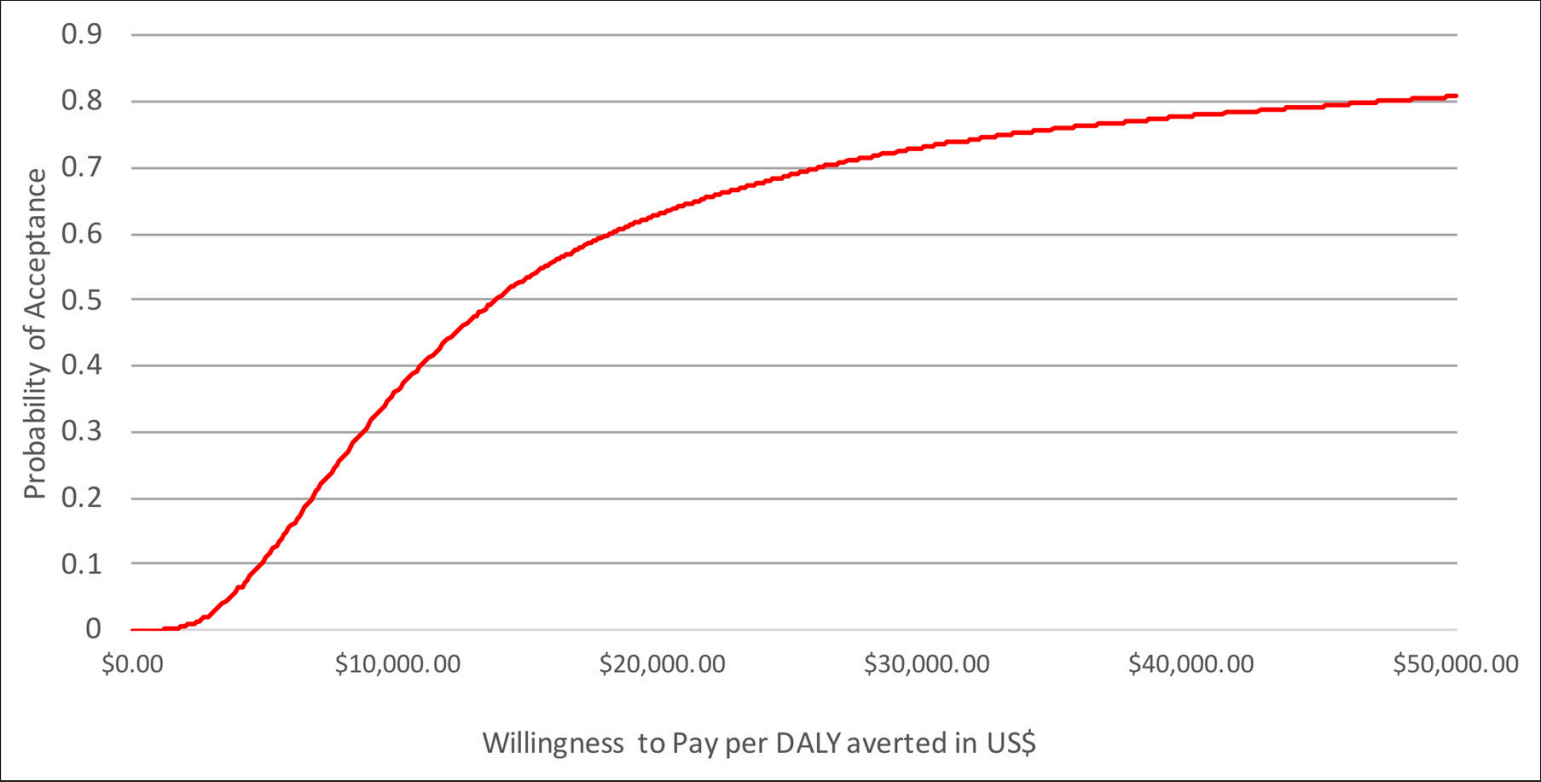
Cost-effectiveness acceptability curve. The X-axis shows a range of increasing cost-effectiveness thresholds, while the Y-axis shows how high the probability is that the screening intervention is cost-effective against a cost-effectiveness threshold when compared to standard care if variance in the data is taken into account. The line shows how high the probability is that the intervention is accepted against a certain cost-effectiveness threshold, given the uncertainty of the parameter estimates.

Figure 2 shows the cost-effectiveness acceptability curve. The curve shows the probability of the screening program being cost-effective (on the y-axis) given a certain cost-effectiveness threshold (on the x-axis). The probability of the intervention being cost-effective increases as the value of the cost-effectiveness threshold increases. Following the curve, the probability that the intervention is successful with a cost-effectiveness threshold of $25,949.85 is 70%.

### Sensitivity analysis

**Figure 3:**
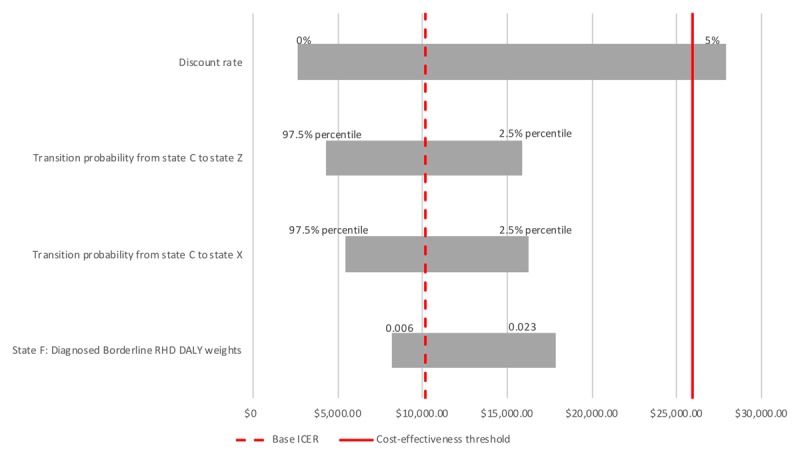
Sensitivity analysis applied to the cost-effectiveness model. The y-axis shows various parameters, while the x-axis shows a range of ICERs. The dotted line shows the ICER of the base model, while the solid line shows the upper cost-effectiveness threshold limit.

Figure 3 shows a tornado plot of the 4 most influential parameters in the sensitivity analysis. The parameter that breaches the cost-effectiveness threshold is the discount rate. Further influential parameters are the rate of transition probabilities from state C: *Untreated Asymptomatic Definite RHD* and state G: *Treated Asymptomatic Definite* to state Z: *Death*, and state C: *Untreated Asymptomatic Definite RHD* to state X: *Surgery*. The least influential parameter presented in the figure is the disutility of knowing that one has borderline RHD, as opposed to not knowing it. The sensitivity to this balance between short term costs and long-term improvement in outcomes is partly a consequence of the high number of borderline RHD cases diagnosed compared to clinically relevant cases. The full table with the results of the sensitivity analysis ordered by magnitude of change of the ICER can be found in the Appendix.

## Discussion

### Results

The results show that implementing a targeted screening of at-risk children is cost-effective in the base model. However, this result is sensitive to parameters which present short-term disutility and costs, and long-term gains in utility, life years gained and costs saved.

Care has to be taken to apply our results in the real world. One of the core challenges in Markov models is estimating the accurate transition probabilities between different states. Only recently (2012) the WHF published an expert consensus for the echocardiographic diagnosis of asymptomatic RHD. For this project, most of the transition probabilities of RHD itself is taken from primary data of a study conducted in Uganda and secondary data from a study conducted in Australia [25,26].

The question is in how applicable this data is for the Brazilian context, because environmental factors are reported to have a big impact on the disease and its course [5]. The main study from which transition probabilities from Asymptomatic Borderline RHD to other states derived was conducted on a sample of children from the cities Kampala and Gulu in Uganda [26]. These areas are believed not to differ too much from the metropolitan context in Brazil. It is unknown, however, how the different contexts of these studies affect the transition probabilities.

Transition probabilities from definite RHD to the other states in this model were mainly based on a study by Roberts et al. [8]. The population density of the study area is 0.2 person per square kilometer. This is significantly different from the Brazilian context, where children affected by RHD live in densely populated metropolitan areas, which could lead to an underestimation of RHD behaviour compared to the densely populated metropolitan context in Brazil. Also, secondary prophylaxis was administered to a part of the participants, which could further depress the transition to more server health states compared to its natural course. However, adherence was so poor that the authors stated that disease course was closer to a natural disease course than to a medically supervised condition. These particularities might explain the underestimation of RHD prevalence of the model and the program’s cost-effectiveness.

The generalizability of the results partly depends on the possibility of differing prevalence between Brazilian states but, currently, information about prevalence of latent RHD is available for only one state [3]. However, adjusted RHD mortality rates across Brazilian states are similar, varying from 7 per 100,000 in Acre to 10 per 100,000 in Rio de Janeiro and Rio Grande do Sul [27]. This indicates the impact of RHD across all Brazilian states.

### Effect of secondary prophylaxis

Only one observational study evaluated the effect of adherence to secondary prophylaxis on heart failure and mortality [28] in a sample of advanced RHD cases. An odds ratio of 3.3 for heart failure and an increase in mortality were observed when a group with poor BPG adherence was compared to those with optimal adherence. It is possible that the preventive effect of secondary prophylaxis on clinical RHD is higher than assumed in this study, meaning that the effectiveness of RHD screening might be underestimated. Conversely, the effect of secondary prophylaxis on progression of Asymptomatic Definite RHD is also uncertain, as large echocardiographic studies are relatively recent. This issue is currently under investigation in the large GOAL (*Gwoko Adunu pa Lutino*; clinicaltrials.gov No. NCT03346525) trial, under enrolment in Uganda. Thus, the reduction modelled in this study may overestimate the benefits of screening.

### Outcome

To estimate the impact of the intervention, an outcome weight that estimates the effect of the intervention on both quality of life and the number of life-years saved was used. No studies estimating QALY weights for different RHD states exist to date, thus we opted to use DALY weights from the GBD 2016 study [18]. Given the sensitivity of the balance between short-term disutility of knowing that one has RHD versus the long term gain in DALYs and YLL averted, future research focussing on estimating specific utility weights for the various stages of disease in this population should be warranted.

### Other cost-effectiveness studies studying screening of RHD

Other previously developed Markov models aiming to assess the cost-effectiveness of RHD screening also dealt with the lack of data about disease course and the effects of secondary prophylaxis on progression, and different solutions were applied to deal with these gaps. Only one study utilized primary data for the Markov model, using the latest available technologies [8], while the other studies derived transition probabilities from secondary data [29,30]. Sensitivity analyses were conducted in these studies to control for these research gaps. The conclusion was that there is a high probability that RHD screening is cost-effective even after sensitivity analysis of key transition probabilities, costs and effect of secondary prophylaxis.

### Other cost-effectiveness studies studying primary prophylaxis

Besides secondary prophylaxis, other effective interventions against RHD exist, being primary prophylaxis – administration of antibiotics to treat streptococcal pharyngitis in order to prevent ARF and consequently RHD – the most impactful one. A meta-analysis by Robertson et al. [9] showed that primary prophylaxis reduces ARF recurrence by 70%. The study also calculates that prevention of one ARF case in South-Africa would cost $46 in 2005. While a formal cost-effectiveness analysis was not conducted in this study, it concludes that there is support for its cost-effectiveness in developing countries, given the low cost to prevent one ARF case compared to treatment costs of ARF and RHD [4]. It is still controversial, however, if primary prophylaxis is preferable and feasible in practice, a question that can only be answered by looking at the local context.

### Successful prevention of RHD in practice

Preventive RHD programs are not only discussed in academia, but also implemented in practice. A successful example is the Cuban RHD program [31]. A mix of primary and secondary prevention of ARF and RHD was introduced for all 5- to 25-year-olds with the goal to reduce morbidity, disability and mortality. A sharp 86.1% decline of direct costs associated with ARF and RHD management was observed, due to the lower number of patients with severe disease, fewer hospitalisations and avoidance of cardiac surgery in later years [31].

### Brazilian context

The Brazilian government decided that for the next 20 years, healthcare spending is frozen and not allowed to grow over the economy [8,23]. An article by Quaglio et al. indicates that austerity measures can have detrimental effects on a populations’ health. However, this effect can be moderated by appropriate investments [32]. Thus, it is crucial for the SUS to make cost-effective decisions.

## Limitations

There were some significant limitations in this study. This first one is that the effects of secondary prophylaxis on the course of Asymptomatic Definite RHD is not well studied, which means that a precise estimation of the effect is missing, especially in the long-term and markedly in adult populations. Thus, creating an ideal lifetime Markov model was deemed to be problematic because of a lack of evidence. Another limitation is the lack of a detailed model of the effect of screening on Asymptomatic Definite RHD, which might lead to an underestimation of the cost-effectiveness of the intervention. This may be highly significant especially for Asymptomatic Definite RHD with more severe echocardiographic findings, a subpopulation with a presumable worse outcome, in which the intervention would have a greater prognostic impact, not included in our model. Furthermore, an exact idea of the administrative costs of the program is missing, which leads to an underestimation of the costs of the intervention. Despite these limitations, to the best of our knowledge this is the first cost-effectiveness analysis of RHD screening in South America, derived from primary screening data in a large population.

## Conclusion

Our model demonstrated that implementing RHD screening programs with handheld echocardiographic machines in Brazilian underserved populations seems to be cost-effective in the base-case scenario. However, given the hard budget constraints imposed by the Brazilian government and the uncertainty around some of the key parameters, future research should focus on studying the effect of secondary prophylaxis, estimating the administrative costs involved in the implementation of comprehensive screening programs and minimizing the disutility of knowing that one individual has borderline RHD.

## Additional File

The additional file for this article can be found as follows:

10.5334/gh.529.s1 Appendix 1. Initial population distribution over states.

## Abbreviations

ARF: Acute Rheumatic Fever

BPG: Benzathine Penicillin G

DALY: Disability-Adjusted Life Years

PROVAR: Programa de RastreamentO da VAlvopatia Reumática

RHD: Rheumatic Heart Disease

SUS: Sistema Único de Saúde

USD: US Dollars

YLL: Years of Life Lost
