# Supplementary material for: Cost-Effectiveness of Rheumatic Heart Disease Echocardiographic Screening in Brazil: Data from the PROVAR+ Study: Cost-effectiveness of RHD screening in Brazil
Source: Glob Heart. 2020 Feb 20;15(1):18. doi: 10.5334/gh.529 (PMC7218764; doi:10.5334/gh.529)
Supplement: Appendix 1. — Initial population distribution over states. [file gh-15-1-529-s1.pdf]

APPENDIX 1:

Initial population distribution over states

An initial cohort is distributed over the different states by a decision tree (Appendix Figure 1). For the standard care cohort, this meant that State A: No RHD contains 97.2% of the population, State B: Undiagnosed Asymptomatic Borderline RHD contains 2.7% of the population and state C: Untreated Asymptomatic Definite RHD contains 0.1% of the population. All other states contain 0%. The distribution across the different states in the screening cohort was informed by the sensitivity of handheld screening [3, 34]. The screening procedure consists of 1. screening and 2. an evaluation of identified positive cases in the hospital (clinical evaluation and echocardiography) to confirm the screening diagnosis. This procedure was assumed to be 69% sensitive in the case of Asymptomatic Borderline RHD and 95% sensitive for Asymptomatic Definite RHD [3, 34]. This meant, that for the screened cohort, the distribution across the states was as follows: State A: No RHD 97.2%, state B: Undiagnosed Asymptomatic Borderline RHD contains 0.837% of the population, state C: Untreated Asymptomatic Definite RHD contains 0.005% of the population, state F: Diagnosed Borderline RHD contains 1.863% of the populations and state G: Treated Asymptomatic RHD contained 0.095% of the population. When a screening is implemented a proportion of the patients will be diagnosed with definite asymptomatic RHD and will receive treatment (clinical follow-up and prophylaxis with BPG when indicated). The difference between the cohort with intervention and the cohort with standard care can be found in the initial distribution of the first cycle. In the patient cohort that models the intervention screened borderline asymptomatic RHD, patients are placed in state F: Diagnosed Asymptomatic Borderline RHD, while in the standard care the unscreened children are placed in state B: Undiagnosed Asymptomatic Borderline RHD. Also, screened

patients are initially placed in state G: Treated Asymptomatic Definite RHD instead of state C: Untreated Asymptomatic Definite RHD.

Transition probabilities

Most of the probabilities are derived from secondary data through analyzing studies. When necessary yearly transition probabilities were calculated by first calculating the rate per year with the following formula:

$$r = - [\ln (1-P)]/t$$

And then recalculating the rate per year into a yearly probability with the following formula:

$$P = 1- \exp (-rt)$$

where P is the probability, r is the rate and t is the time period of interest [35].

The subclinical (asymptomatic) disease states were based on the WHF criteria of 2012 [12]. The clinical disease states were based on the various states described by the Australian RHD guidelines [13], based on the study Roberts et al. [8]. Assumptions were made for transition probabilities that could not be derived from studies. An overview of the studies used can be found in Appendix Table 1. The model itself consists of two main pathways, as shown in Figure 1. The green pathway represents the course of RHD of a patient when the patient is under treatment of the public health system, while the orange pathway shows the natural course of disease when the patient is not under treatment or clinical follow-up.

The probability from state A: No RHD to state B: Undiagnosed Asymptomatic Borderline RHD is estimated by using the following formula [36]:

Incidence rate = prevalence / average duration disease

A couple of assumptions need to be made for the use of this formula. The first assumption is that the disease in the target population is in a steady state; prevalence remains stable over a longer period of time and the inflow and outflow of diseased are equal. While the study by Nascimento et al. shows this is not the case [3], the peak prevalence of the study (49 per 1.000 children for borderline cases and 9.3 per 1,000 for definite cases) was assumed to equal stable prevalence. The second assumption is that the average duration of disease for Asymptomatic Borderline RHD is estimated to be approximately 3.63 years, and the average duration of disease for asymptomatic definite RHD 10.72 years. This assumption is estimated by using the yearly probability of staying in state B: Undiagnosed Asymptomatic Borderline RHD and respectively state C: Asymptomatic Definite RHD and calculate at what time more than 99.5% of a hypothetical cohort stopped being affected by their respective affliction of RHD (disease could have either regressed to no RHD or progressed to worse health states, such as definite RHD). Then the average duration of disease time was calculated for 99.5% of this hypothetical cohort.

For adults above 20 years old it was assumed that the probability of being healthy to getting Asymptomatic Definite RHD is halved. Furthermore, since it is by definition not possible for adults to have Asymptomatic Borderline RHD, the pathway to this state was cut for this group. Lastly, the proportion of the population with the age of 19 that was still in the Asymptomatic Borderline RHD was transferred to state A: No RHD in the Markov model.

Almost all the transition probabilities for state B: Undiagnosed Asymptomatic Borderline RHD to various states were derived from primary survival data from a study conducted in Uganda by Beaton et al. [26] using the msSurv package for the statistical program R [37]. The disease course of asymptomatic borderline cases from this study are

equal to the disease course of Asymptomatic Borderline cases in this model. The transition probabilities were calculated by first calculating the probability of transitioning to different states between the start of the study and the third year, and then transforming that probability to yearly probabilities. The transition probability from state B: Undiagnosed Asymptomatic Borderline RHD to state Z: Death was assumed to be equal to State A: No RHD to state Z: Death. The reason for this is that the data showed a lower mortality rate than the standard mortality rate for a healthy individual in this model, which was deemed unrealistic. To control for the variance of state B: Undiagnosed Borderline RHD a dirichlet distribution was used. This distribution was centered on the yearly probabilities calculated above. To calculate parameters for the probabilistic sensitivity analysis a population size of 162 was used, which is the sample size of borderline RHD patients in the study by Beaton et al.

The study by Cannon et al. [25] was used to determine the transition probabilities from state C: Untreated Asymptomatic Definite RHD to state A: No RHD, state B: Undiagnosed Asymptomatic Borderline RHD, state C: Untreated Asymptomatic Definite RHD, state D: Untreated Mild Clinical RHD, state E: Untreated Severe RHD and state Z: Death. Cannon et al. use the Australian RHD guidelines [38] for their states. It was assumed that the Asymptomatic Definite RHD ceases identified in the study by Beaton et al. [26] were similar to the mild RHD cases identified in the paper by Cannon et al. [25]; in both cases one can speak of an asymptomatic, clinically trivial to mild valve disease, only with findings in auscultation. Under this assumption, it was possible to 'connect' both the databases as there is an overlap of Asymptomatic Definite RHD in both the Beaton et al. study [26] and the Cannon et al. study [25]. Yearly transition probabilities were calculated by using the formula mentioned at the start of the Appendix to estimate yearly rates based on 10-year transition

probabilities, and then recalculate those rates to yearly transition probabilities. The transition probabilities for State D: Untreated Mild Clinical RHD are based on the moderate state in the study by Cannon et al. [25]. Transition probabilities for state E: Untreated Severe RHD were based on the severe state of the study [25]. However, given that the patients in the study by Cannon et al. [25] were under treatment, the transition probability from state E: Untreated Severe RHD to state X: Surgery was adjusted to mimic the natural discourse. This number was calculated based a study by Albuquerque et al. [39]. As an estimate, it was assumed that because of medication non-adherence, for every 70 patients an additional 30 patients need surgery, given that 30% of the heart failure decompensation could be attributed to medication non-adherence by the patients.

The treated RHD track starts with state F: Diagnosed Asymptomatic Borderline RHD. This state represents the proportion of asymptomatic borderline RHD patients diagnosed by screening. The treatment of borderline RHD patients consists of a yearly cardiologic check-up which consists of a cardiology consult and an echocardiogram. No BPG is administered in this group. It is assumed that everybody adheres to the yearly check-up. Because of this yearly check-up, it is assumed that any individual with a progression in RHD is automatically transferred to state G: Treated Asymptomatic Definite RHD because of early detection. This assumption is based on data by Beaton et al. [26], which shows that most progression happens between the first and second year. The transition probability between state F and stage G is assumed to be equal to the transition probability between state B: Undiagnosed Asymptomatic Borderline RHD and state C: Untreated Asymptomatic Definite RHD. From stage G: Treated Asymptomatic Definite RHD it is possible to transition to state H: Treated

Mild Clinical RHD and state R: Resolved RHD. State H: Treated Mild Clinical RHD is linked to State X: Surgery and state I: Treated Severe Clinical RHD.

From state G: Treated Asymptomatic Definite RHD and state H: Treated Mild Clinical RHD, to state H: Treated Mild Clinical RHD and state I: Treated Severe Clinical RHD the effect of every three-week intramuscular BPG injection as secondary prophylaxis is modeled. It is assumed that with full adherence progression in RHD is stopped between state G: Treated Asymptomatic Definite RHD and state H: Treated Mild Clinical RHD, and halved between state H: Treated Mild Clinical RHD and state I: Treated Severe RHD. This assumptions were made earlier in the study of Cannon et al. [25]. It was assumed that secondary prophylaxis did not have any further effects on disease progression and mortality.

Transition probabilities from state C: Untreated Asymptomatic Definite RHD to state H: Treated Mild Clinical RHD and state D: Untreated Mild Clinical RHD, and transition probabilities from state D: Untreated Mild Clinical RHD to state I: Treated Severe Clinical RHD and state E: Untreated Severe Clinical RHD model the adherence to medication. A study by Pelajo et al. [40] shows that 65% of the children adhere to secondary prophylaxis in asymptomatic RHD, while a study by Mussi et al. [41] shows that in standard care 60% of the symptomatic participants adhered to their medication. In other words, 65% of the total transition from state C: Untreated Asymptomatic Definite RHD to Clinical RHD goes to state H: Treated Mild Clinical RHD and 60% of the total transition probability from state D: Untreated Mild Clinical RHD to severe RHD goes to state I: Treated Severe Clinical RHD. It was assumed that once patients adhered to a certain treatment that they would continue to adhere to further treatment if their diseased progressed. As an example, this means that all the patients for which the disease progresses in state H: Treated Mild Clinical RHD go to state I:

Treated Severe Clinical RHD. This assumption was made because once the decision between adherence and non-adherence was made, people did not seem to change their minds as can be seen in the follow-up study by Mussi et al. [41].

State X: Surgery represents the proportion of the population in which RHD has developed to such an extent that surgery is needed. The yearly mortality risk of surgery (the risk of dying before, during and after the operation during one year) is derived from the study by Xavier et al. (Medium-term outcomes of 78,808 patients after heart valve surgery in a middle-income country: A nationwide population-based study). State X: Surgery is modelled in such a way that when a patient gets surgery, he can only stay for one year in this state. The reason for modelling a one-year transition state as opposed to an immediate surgery is the waiting times for surgery in the public health system in Brazil [42].

Transition probabilities from state K: Post Surgery to state Z: Death and state X: Surgery were derived from a study by Ribeiro et al. [43]. It was assumed that after surgery you stayed in a state that yielded high cost and disutility, because of medical consults, medicine and a not fully functioning heart. Patients in this state are also at risk of one or multiple resurgeries, which is reflected in the transition probability from state K: Post Surgery to state X: Surgery.

All the transition probabilities from state F: Diagnosed Asymptomatic Borderline RHD (to state A: No RHD, to state G: Treated Mild Clinical RHD and state Z: Death) were assumed to be equal to those of state B: Undiagnosed Asymptomatic Borderline RHD, since the annual check-up itself does not influence the course of disease. However, because of the annual check-up, it was assumed that patients are diagnosed the moment that borderline RHD becomes definite RHD and thus stay in the healthcare system. In the model this means that

patients who are diagnosed with definite RHD from state F: Diagnosed Asymptomatic Borderline RHD will go straight to state G: Treated Asymptomatic Definite RHD (Definite plus clinically trivial disease with mild valve involvement).

External validation

The model predicted a prevalence of Asymptomatic Definite RHD of 2.26 per 1,000 for the age group 11 to 13.9 and a prevalence of Asymptomatic Definite RHD of 6.36 per 1,000 for the age group of 14 to 17.9 in the target population. Observed prevalence [3) in these age groups were respectively 3.1 per 1,000 in the age group 11 to 13.9 and 9.3 per 1,000 in the age group 14 and older. The predicted prevalence of Asymptomatic Borderline RHD in the age groups 11 to 13.9 and 14 to 17.9 were respectively 33.62 per 1,000 children and 45.63 per 1,000 children. Observed prevalence [3] for these age groups were respectively 35 per 1,000 for the age group 11 to 13.9 and 49 per 1,000 for the age group 14 and older.

Costs

Costs for state R: Resolved RHD depend on from which state the specific patient comes from. Patients coming from state G: Treated Asymptomatic Definite (subclinical definite and clinically trivial RHD with mild valve involvement) RHD have different costs associated with them compared to state H: Treated Mild Clinical RHD.

For the intervention, it is assumed that 75 images are made per day, which amounts to 9.375 images per hour assuming an eight-hour workday. In these five years, it was assumed that the screening could be conducted for an 8.5-month period per year, when one takes into account the school holidays in Brazil and other national free days. It was assumed that in a week, the team could effectively screen for six hours per day for five days. By multiplying the total amount of work hours per year with the number of images made per hour (1,635

work hours per year, 9.375 images per hour) a total of 15,236 scans were assumed to be made per year.

In order to acquire this number of images, a team of two research nurses, an imagining technician, a biomedical technician and a physician (echo expert) are required. Assuming 4.35 weeks per month, the monthly salaries were divided by the number of hours worked (assuming a 40 hour work week) per week to calculate the salary cost per hour. This number was divided by the number of scans per hour to calculate the salary cost per scan. This cost was estimated to be $6.26.

The equipment costs to support this number of images per day are two machines and a laptop, with a lifetime of five years and no resell value after those five years. The fixed costs per year were divided by the number of scans per year to calculate the fixed costs per scan, which is $0.34. Adding up the salary costs per scan and the fixed costs per scan, one scan costs a rounded total of $6.60.

All of the cases with a positive handheld screening result will be referred to a cardiologist for further screening. There, they will get an echocardiogram and a cardiology consult. Cases diagnosed with definite RHD will receive penicillin. Due to the specificity of handheld screening (65%) [44], a proportion of the population will get a false-positive diagnosis. These cases will be evaluated as well, which results in extra costs. These costs, which are similar to cost of follow up for diagnosed borderline RHD are presented in Appendix Table 3 for false-positive and borderline RHD cases and Appendix Table 4 for definite RHD cases.

The variance of the cost parameters is based on the variance of the cost of surgery. Given that surgery is relative complex compared to the other treatments, it can be expected

that this variance is higher than the variance of the other treatments. This might result in an over-estimation of the uncertainty the costs of treatments other than surgery.

Sensitivity analysis

The sensitivity of the base-case treatment adherence (65%) for the treatment of asymptomatic definite RHD was tested by decreasing the adherence to 33% and increasing it to 100%. The sensitivity of treatment adherence of severe clinical RHD (base case 60%) was tested by decreasing the parameter to 30% and increasing it to 100%. A table of the base-case assumption and the sensitivity analysis around the base case can be found in Appendix Table 8. It was assumed that there was no loss to follow-up after surgery.

The full results of the one-way sensitivity analysis of the parameters can be found in Appendix Table 9. The results are ordered based on the magnitude of change; the parameters that showed the biggest change are at the top. An explanation about how the sensitivity analysis was conducted can be found in the methods section.

Appendix Figure 1.

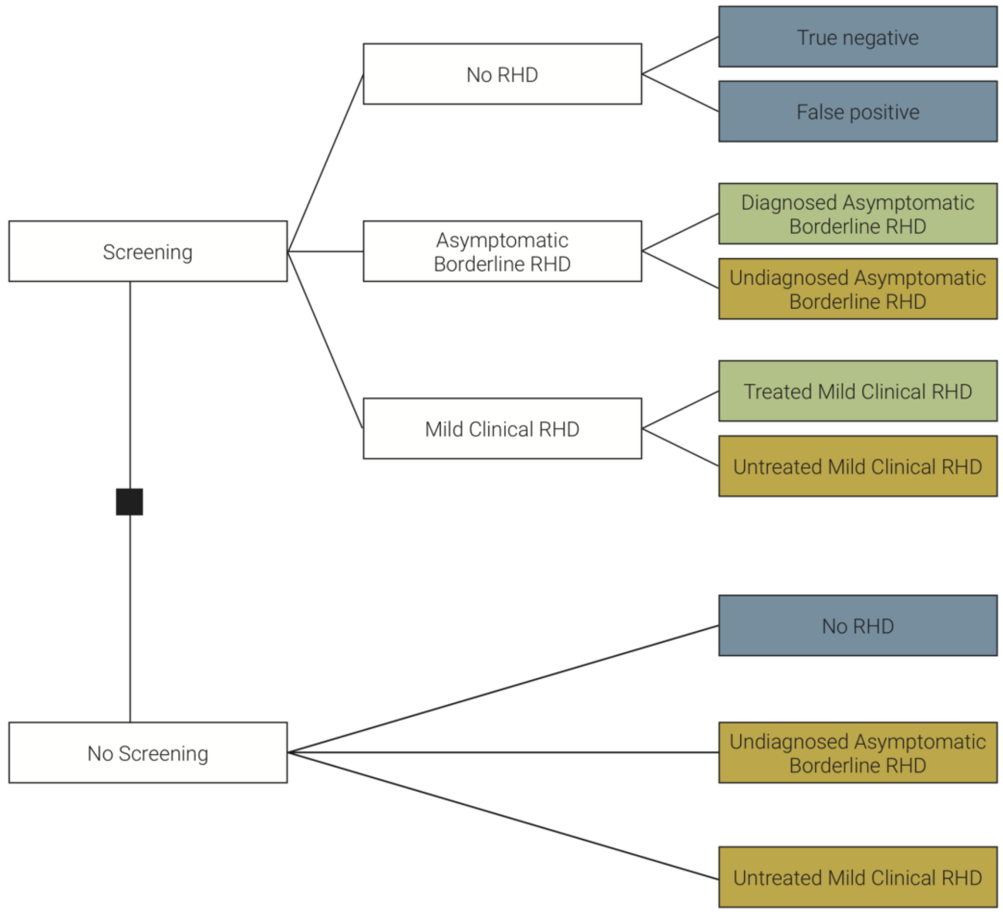

Appendix Table 1. Transition probabilities

Name:	Trans. Prob.	Source:	Distribution used for probabilistic analysis:	Distribution parameters (α for dirichlet):
tpA2A	0.9839348	1-tpA2B+-tpA2C+-tpA2Z	Dirichlet	5900
tpA2B	0.0136975	Nascimento et al. 2016 [3]	Dirichlet	82
tpA2C	0.0008675	Nascimento et al. 2016 [3]	Dirichlet	5
tpA2Z	0.0015	Neves and Garcia 2015 [45]	Dirichlet	9
tpA2A, age 20+	0.9984122	1-tpA2b20+-tpA2C20+-tpA2Z20+	Dirichlet	5986
tpA2B, age 20+	0	Borderline RHD is by definition non-existent in adults	Dirichlet	0
tpA2C, age 20+	0.0000878	Assumed, ½ tpA2C	Dirichlet	1
tpA2Z	0.0015	Neves and Garcia 2015 [45]	Dirichlet	9
tpB2A	0.2485657	Primary data Beaton et al	Dirichlet	40.27
tpB2B	0.7255101	1-tpB2A-tpB2C-tpB2D-tpB2H-tpB2Z	Dirichlet	116.15
tpB2C	0.0159187	Primary data Beaton et al	Dirichlet	2.58
tpB2D	0.0029769	Primary data Beaton et al, times 1- adherence mild RHD	Dirichlet	1.38
tpB2H	0.0055285	Primary data Beaton et al, times adherence mild RHD	Dirichlet	1.38
tpB2Z	0.0015	Neves and Garcia 2015 [45]	Dirichlet	0.24
tpC2R	0.005430818	Cannon, Roberts, and Milne 2017 [25]	Dirichlet	1.81

Name:	Trans. Prob.	Source:	Distribution used for probabilistic analysis:	Distribution parameters (α for dirichlet):
tpC2B	0.0542572	Cannon, Roberts, and Milne 2017 [25]	Dirichlet	18.12
tpC2C	0.9070961	1-tpC2R-tpC2B-tpC2C-tpC2D-tpC2E-tpC2H-tpC2I-tpC2X-tpC2Z	Dirichlet	296.29
tpC2D	0.007003056	Cannon, Roberts, and Milne 2017 [25], times 1 –adherence mild RHD	Dirichlet	6.68
tpC2E	0.002594429	Cannon, Roberts, and Milne 2017 [25], times 1- adherence severe RHD	Dirichlet	2.17
tpC2H	0.013005676	Cannon, Roberts, and Milne 2017 [25], times adherence mild RHD	Dirichlet	6.68
tpC2I	0.003891644	Cannon, Roberts, and Milne 2017 [25], times adherence severe RHD	Dirichlet	2.17
tpC2X	0.005220971	Cannon, Roberts, and Milne 2017 [25]	Dirichlet	1.74
tpC2Z	0.0015	Neves and Garcia 2015 [45]	Dirichlet	0.50
tpD2R	0.003453169	Cannon, Roberts, and Milne 2017 [25]	Dirichlet	0.56
tpD2B	0.013812674	Assumed, tpD2R*4	Dirichlet	2.22

Name:	Trans. Prob.	Source:	Distribution used for probabilistic analysis:	Distribution parameters (α for dirichlet):
tpD2C	0.040700112	Cannon, Roberts, and Milne 2017 [25]	Dirichlet	6.55
tpD2D	0.908617282	1-tpD2R-tpD2B-tpD2C-tpD2E-tpD2I-tpD2X-tpD2Z	Dirichlet	146.29
tpD2E	0.008433976	Cannon, Roberts, and Milne 2017 [25], times 1- adherence severe RHD	Dirichlet	2.26
tpD2I	0.005622651	Cannon, Roberts, and Milne 2017 [25], times adherence severe RHD	Dirichlet	2.26
tpD2X	0.016933816	Cannon, Roberts, and Milne 2017 [25]	Dirichlet	2.73
tpD2Z	0.002426321	Cannon, Roberts, and Milne 2017 [25]	Dirichlet	0.39
tpE2R	0.000601626	Cannon, Roberts, and Milne 2017 [25]	Dirichlet	0.06
tpE2C	0.005746338	Cannon, Roberts, and Milne 2017 [25]	Dirichlet	0.55
tpE2D	0.0072308	Cannon, Roberts, and Milne 2017 [25]	Dirichlet	0.69
tpE2E	0,8390769	1-tpE2R-tpE2B-tpE2C-tpEE2D-tpEE2X-tpE2Z	Dirichlet	80.55

Name:	Trans. Prob.	Source:	Distribution used for probabilistic analysis:	Distribution parameters (α for dirichlet):
tpE2X	0.133966784	Assumed, tpI2X plus extra patients due to inadherence [39]	Dirichlet	12.86
tpE2Z	0.013377209	Cannon, Roberts, and Milne 2017 [25]	Dirichlet	1.28
tpF2A	0,2485657	Equal to tpB2A		
tpF2F	0,7255101	1-tpF2A-tpF2G-tpF2Z		
tpF2G	0,0244242	Equal to tpB2C+tpB2D+tpB2H		
tpF2Z	0.0015	Equal to tpB2Z		
tpG2G	0,938811905	1-tpG2H-tpG2R-tpG2Z		
tpG2H	0	tpC2H*effect secondary prophylaxis (100% reduction)		
tpG2R	0,059688095	Equal to tpC2R+tpC2B		
tpG2Z	0.0015	Equal to tpC2Z		
tpH2H	0.964812615	1-tpH2I-tpH2R-tpH2Z-tpH2X		
tpH2I	0.007028313	Equal to tpD2E*effect secondary prophylaxis (50% reduction)		
tpH2R	0.017265843	Equal to tpD2R+tpD2B		
tpH2X	0.008466908	Equal to tpD2X		
tpH2Z	0.002426321	Equal to tpD2Z		
tpI2I	0.892110217	1-tpI2R-tpI2X-tpI2Z		

Name:	Trans. Prob.	Source:	Distribution used for probabilistic analysis:	Distribution parameters (α for dirichlet):
tpI2R	0.000601626	Equal to tpE2R + tpE2B		
tpI2X	0.093910947	Equal to E2X without extra patients		
tpI2Z	0.013377209	Equal to E2Z		
tpR2R	0.9985	1-tpR2Z		
tpR2Z	0.0015	Assumed, Neves and Garcia 2015 [45]	Beta	α = 8.99, β = 5,987.01
tpK2K	0.989465544	1-tpK2X-tpK2Z		244.40
tpK2X	0.000283491	Ribeiro et al [43]	Dirichlet	0.07
tpK2Z	0.010250965	Ribeiro et al [43]	Dirichlet	2.53
tpX2K	0.788	1-tpX2Z		
tpX2Z	0.212	Preoperative mortality due to waiting times: 11.3%, assumed [42]<br>Perioperative risk: 96.37% [46]<br>one-year post-operative survival: 93.73% [46]	Beta	α = 8,978.62, β = 33,373.38

Footnote: tp stands for the yearly transition probability to a different state. The capital letter stands for the state. 2 means ´to´. The table should thus be read as follows: the transition probability from state A to Z is 0.0015 (tpA2Z is 0.0015).

Appendix Table 2. Cost of screening per hour

Unit per team and equipment:	Quantity:
Cases screened per day (experience from PROVAR study)	75
Cases screened per hour (Assumed 8 hour work day)	9.375
Readings of screens per hour by Physician (echo expert)	12
Assumed screens per year (no school in December, January and July, carnival and national holidays)	10388
**Item:**	**Cost per hour:**
**Hourly Salaries**	
2x research nurse	R$40.92
Imaging technician	R$15.65
Biomedical technician	R$17.56
Physician (echo expert)	R$73.90
**Total salary cost per scan**	R$20.63 ($6.26)
**Equipment costs per year:**	
Cost of 2 handheld screening machines	$16 000.00
Cost laptop	$1500.00
**Yearly cost (5-year lifetime of equipment)**	3 300.00
**Total fixed cost per scan (Yearly costs divided**	$0.34

**by yearly cases screened)**	
**Total cost per scan (salary plus fixed)**	$6.60

The source of the costs is primary data from the PROVAR project.

Appendix Table 3. Cost of diagnosed borderline RHD

Item:	Cost:
1x echocardiogram	R$39.94
1x cardiology consult	R$45.23
**Total**	R$85.17 ($25.84)

The source of the costs is primary data from the PROVAR project.

Appendix Table 4. Cost of treatment of definite asymptomatic RHD (state G)

Item:	Cost:
12x 21-day secondary prophylaxis penicillin	R$39.94
1x GP consult	R$74.88
1x cardiology consult	R$45.23
1x echocardiogram	R$39.94
**Total**	R$309.57 ($93.93)

The source of the costs is primary data from the PROVAR project.

Appendix Table 5. Cost of treatment of definite symptomatic RHD (state H)

Item:	Cost:
12x 21-day secondary prophylaxis penicillin	R$39.94
1x GP consult	R$74.88
1x cardiology consult	R$45.23
1x echocardiogram	R$39.94
Treatment cardiac impairment or Carditis	R$666.91
**Total**	R$1,112.17 ($337.47)

The source of the costs is primary data from the PROVAR project and DataSUS. Weighted average of expenditure on procedure codes 0303060115 and 0303060123 between 2014–2016 (end date 29/04/2016).

Appendix Table 6. Cost of surgery (state X)

Item:	Cost:
Treatment of aortic valve stenosis	R$10,869.42
Treatment of pulmonary valve stenosis.	R$13,268.40
Correction of tricuspid regurgitation	R$16,491.46
Ross procedure	R$15,381.90
Valvar prosthesis implant	R$13,137.01
Tricuspid valvuloplasty	R$22,180.48
Surgical valvuloplasty	R$22,180.48
Valvuloplasty, multiple valves	R$15,172.21
**Weighted average**	R$13,579.78 ($4,120.51)

The source of the costs is primary data from the DataSUS, based on disease codes presented in do Espírito Santo Freire et al. [33]. Timeframe of the data evaluation was between September 2014 and December 2016.

Appendix Table 7. DALY weights for different health states

State:	DALY weight:	95% UI:	GBD 2016 Sequelae[18]:
Stata A: No RHD	0.000	-	-
State B: Undiagnosed Asymptomatic Borderline RHD	0.000	-	-
State C: Untreated Asymptomatic RHD	0.041	0.026 – 0.062	Asymptomatic and mild heart failure due to rheumatic heart disease
State D: Untreated Mild Clinical RHD	0.049	0.031 – 0.072	Rheumatic heart disease, without heart failure
State E: Untreated Severe Clinical RHD	0.179	0.122 – 0.251	Severe heart failure due to rheumatic heart disease
State F: Diagnosed Asymptomatic Borderline RHD	0.012	0.006 – 0.023	Generic uncomplicated disease: anxiety about diagnosis
State G: Treated Asymptomatic RHD	0.041	0.026 – 0.062	Asymptomatic and mild heart failure due to rheumatic heart disease
Stage H: Treated Mild Clinical RHD	0.049	0.031 – 0.072	Rheumatic heart disease, without heart failure
State I: Treated Severe Clinical RHD	0.179	0.122 – 0.251	Severe heart failure due to rheumatic heart disease
State R: Resolved RHD	0.012	0.006 – 0.023	Generic uncomplicated disease: anxiety about diagnosis

State	DALY weight	95% UI	GBD 2016 Sequela [18]
State K: Post Surgery	0.072	0.047 – 0.103	Moderate heart failure due to rheumatic heart disease

State X: Surgery	0.179	0.122 – 0.251	Severe heart failure due to rheumatic heart disease
State Z: Death	1	-	-

Abbreviations: CI: confidence interval; RHD: Rheumatic Heart Disease. Obs.: Asymptomatic RHD refers to asymptomatic definite RHD plus mild valve involvement with clinically trivial significance.

Appendix table 8. Medication adherence base case and sensitivity analysis.

Treatment adherence	Base adherence	Low adherence	High adherence
Mild clinical RHD	65%	33%	100%
Severe Clinical RHD	60%	30%	100%

Abbreviations: RHD: Rheumatic Heart Disease.

Appendix Table 9. Results of the one-way sensitivity analysis

Parameter Name:	Low:	High:	Difference:
Discount rate	$2,626.98	$27,877.47	$25,250.49
tpC2Z	$15,904.93	$4,290.27	$11,614.66
tpC2X	$16,234.28	$5,443.53	$10,790.74
Disutility state F	$8,211.98	$17,876.42	$9,664.44
tpB2C	$15,239.64	$6,534.24	$8,705.40
tpB2H	$16,962.67	$8,735.00	$8,227.66
tpD2Z	$13,140.10	$5,192.65	$7,947.45
Cost of state X	$10,198.62	$3,377.55	$6,821.07
Disutility state G	$8,301.28	$14,740.09	$6,438.80
tpK2Z	$13,694.63	$7,310.66	$6,383.97
tpJ2Z	$8,468.26	$13,909.09	$5,440.83
Cost of screening	$9,659.82	$14,449.76	$4,789.95
tpD2X	$12,315.76	$7,578.14	$4,737.62
tpC2I	$13,538.32	$9,232.36	$4,305.96
Effect prophylaxis on Asymptomatic Definite RHD	$8,977.69	$12,986.35	$4,008.67
Cost of state K	$10,245.79	$6,302.30	$3,943.49
tpB2D	$13,291.55	$9,357.01	$3,934.54
Disutility state C	$12,033.56	$8,322.96	$3,710.60
Cost of state F	$9,645.84	$6,177.25	$3,468.59
tpA2Z	$11,746.46	$8,560.16	$3,186.30
tpC2B	$8,751.12	$11,924.39	$3,173.27
Sensitivity screening Asymptomatic Borderline RHD	$11,914.09	$9,344.36	$2,569.73

Parameter Name	Low	High	Difference
Effect prophylaxis on Mild Clinical RHD	$11,652.47	$9,089.97	$2,562.50
tpD2B	$9,300.98	$11,811.43	$2,510.45
tpC2E	$12,006.28	$9,574.90	$2,431.39
Disutility state R	$9,451.06	$11,735.88	$2,284.81
tpC2H	$11,298.19	$9,613.74	$1,684.45
tpB2Z	$10,426.89	$8,787.34	$1,639.55
tpB2A	$9,383.49	$10,953.44	$1,569.95
Cost of state H	$10,216.17	$8,927.77	$1,288.39
tpD2I	$11,100.34	$9,814.04	$1,286.30
Cost of state I	$10,178.10	$8,975.04	$1,203.06
tpD2A	$9,898.84	$11,099.02	$1,200.18
tpE2Z	$10,555.68	$9,432.43	$1,123.25
Disutility state H	$10,665.75	$9,556.08	$1,109.67
tpC2A	$9,803.85	$10,883.32	$1,079.46
Sensitivity screening Asymptomatic Definite RHD	$10,924.53	$9,914.51	$1,010.02
tpD2C	$9,695.95	$10,672.40	$976.45
tpC2D	$10,755.42	$9,848.94	$906.49
Disutility state K	$10,552.10	$9,688.74	$863.36
tpD2E	$10,723.51	$9,938.52	$784.98
Disutility state D	$10,425.94	$9,814.53	$611.40
Disutility state I	$10,425.84	$9,818.34	$607.50
Adherence Severe Clinical RHD treatment	$10,317.01	$9,926.07	$390.94
tpE2X	$10,354.68	$10,025.30	$329.38

Parameter Name	Low	High	Difference
Cost of state R, coming from state H	$10,163.76	$9,871.57	$292.18
Cost of state G	$10,044.54	$10,334.89	$290.36
tpE2C	$10,088.46	$10,356.95	$268.49
tpE2A	$10,125.28	$10,381.95	$256.67
Cost of state R, coming from state G	$10,057.99	$10,310.72	$252.73
tpE2D	$10,086.15	$10,335.64	$249.49
tpX2Z	$10,272.46	$10,026.63	$245.83
Disutility state E	$10,251.10	$10,021.55	$229.56
Disutility state X	$10,243.90	$10,030.25	$213.65
tpK2X	$10,165.32	$9,987.01	$178.31
Adherence Mild Clinical RHD treatment	$10,125.14	$10,175.91	$50.77
tpA2C, 20+ years old	$10,152.38	$10,137.68	$14.70
Cost of state R, coming from state I	$10,148.59	$10,140.37	$8.22
tpA2C	$10,151.05	$10,144.29	$6.76
Incidence rate adult RHD	$10,150.44	$10,144.28	$6.17
tpA2Z	$10,146.79	$10,150.59	$3.80
tpA2B	$10,148.82	$10,147.90	$0.92

Abbreviations: RHD: Rheumatic Heart Disease.

References

1. **Watkins DA, Johnson CO, Colquhoun SM,** et al. Global, Regional, and National Burden of Rheumatic Heart Disease, 1990–2015. New England Journal of Medicine. 2017;377(8):713–22.

2. **Meira ZMA, Goulart EMA, Colosimo EA, Mota CCC.** Long-term Follow-up of Rheumatic Fever and Predictors of Severe Rheumatic Valvar Disease in Brazilian Children and Adolescents. Heart. 2005;91(8):1019–22.

3. **Nascimento BR, Beaton AZ, Nunes MCP,** et al. Echocardiographic Prevalence of Rheumatic Heart Disease in Brazilian Schoolchildren: Data from the PROVAR Study. International Journal of Cardiology. 2016;219:439–45.

4. **Terreri MT, Ferraz MB, Goldenberg J, Len C, Hilário MO.** Resource Utilization and Cost of Rheumatic Fever. Journal of Rheumatology. 2001;28(6):1394–7.

5. **Marijon E, Mirabel M, Celermajer DS, Jouven X.** Rheumatic Heart Disease. Lancet. 2012;379(9819):953–64.

6. ARF RHD Guideline. 2016:55-8.

7. **Barbosa PJB, Mülle RE.** Diretrizes Brasileiras para diagnóstico, tratamento e prevenção da febre reumática. Arq Bras Cardiol. 2009;93(Suppl 4):127–47.

8. **Roberts K, Cannon J, Atkinson D,** et al. Echocardiographic Screening for Rheumatic Heart Disease in Indigenous Australian Children: A Cost–Utility Analysis. Journal of the American Heart Association. 2017;6(3):e004515.

9. **Robertson KA, Volmink JA, Mayosi BM.** Antibiotics for the Primary Prevention of Acute Rheumatic Fever: A Meta-Analysis. BMC Cardiovascular Disorders. 2005;5(1):11.

10. **Nascimento BR, Sable C, Nunes MCP,** et al. Comparison Between Different

Strategies of Rheumatic Heart Disease Echocardiographic Screening in Brazil: Data From the PROVAR (Rheumatic Valve Disease Screening Program) Study. Journal of the American Heart Association. 2018;7(4).

11. **Sato RC, Zouain DM.** Markov Models in health care. Einstein (Sao Paulo). 2010;8(3):376–9.

12. **Reményi B, Wilson N, Steer A,** et al. World Heart Federation criteria for Echocardiographic Diagnosis of Rheumatic Heart Disease: an Evidence-Based Guideline. Nature Reviews Cardiology. 2012;9(5):297–309.

13. **Brandner A, Schreiweis B, Aguduri LS,** et al. The Patient Portal of the Personal Cross-Enterprise Electronic Health Record (PEHR) in the Rhine-Neckar-Region. In: Hoerbst A, Hackl WO, De Keizer N, editors. Exploring Complexity in Health: An Interdisciplinary Systems Approach. 228: IOS Press; 2016. p. 157–61.

14. XE Currency Converter - Live Rates 1145 Nicholson Rd, Suite 200 Newmarket ON, L3Y 9C3 Canada: XE.com; [Available from: http://www.xe.com/currencyconverter/.

15. XE Currency Converter - Live Rates Newmarket, ON, Canada: XE.com; 2017 [Available from: http://www.xe.com/currencyconverter/.

16. Sisbacen®. Calculadora do Cidadão Brasília: Banco Central do Brasil 2017 [Available from: https://www3.bcb.gov.br/CALCIDADAO/publico/corrigirPorIndice.do?method=corrigirPorIndice.

17. **Ribeiro RA, Mello RGB, Melchior R,** et al. Annual Cost of Ischemic Heart Disease in Brazil: Public and Private Perspective. Arquivos Brasileiros de Cardiologia. 2005;85(1):3–8.

18. **Network GBoDC.** Global Burden of Disease Study 2016 (GBD 2016) Disability Weights. Seattle, United States: Institute for Health Metrics and Evaluation (IHME); 2017.

19. **Bradley-Hewitt T, Dantin A, Ploutz M,** et al. The Impact of Echocardiographic Screening for Rheumatic Heart Disease on Patient Quality of Life. The Journal of Pediatrics. 2016;175:123–9.

20. **Szwarcwald CL, Corrêa da Mota J, Damacena GN, Sardinha Pereira TG.** Health inequalities in Rio de Janeiro, Brazil: Lower healthy life expectancy in socioeconomically disadvantaged areas. American Journal of Public Health. 2011;101(3):517–23.

21. **Baltussen RM, Adam T, Tan-Torres Edejer T,** et al. Making Choices in Health: WHO Guide to Cost-Effectiveness Analysis. 2003.

22. **Manyemba J, Mayosi BM.** Penicillin for secondary prevention of rheumatic fever. Cochrane Database of Systematic Reviews. 2002 (3).

23. **Soarez PCD, Novaes HMD.** Cost-Effectiveness Thresholds and the Brazilian Unified National Health System. Cadernos de Saúde Pública. 2017;33(4):e00040717.

24. **Prado CCL.** A adoção do limite custo-efetividade na incorporação de tecnologias no SUS–o que se pode esperar. Revista Eletrônica Gestão e Saúde. 2015(4):3127–49.

25. **Cannon J, Roberts K, Milne C, Carapetis JR.** Rheumatic Heart Disease Severity, Progression and Outcomes: A Multi-State Model. Journal of the American Heart Association. 2017;6(3).

26. **Beaton A, Aliku T, Dewyer A,** et al. Latent Rheumatic Heart Disease: Identifying the Children at Highest Risk of Unfavorable Outcome. Circulation. 2017;136(23):2233–44.

27. **Brant L, Nascimento B, Passos V,** et al. Variations and particularities in cardiovascular disease mortality in Brazil and Brazilian states in 1990 and 2015: Estimates

from the Global Burden of Disease. Rev Bras Epidemiol. 2017;20(suppl 1):116–28.

28. **Okello E, Longenecker CT, Beaton A, Kamya MR, Lwabi P.** Rheumatic heart disease in Uganda: Predictors of morbidity and mortality one year after presentation. BMC Cardiovascular Disorders. 2017;17(1):20.

29. **Zachariah JP, Samnaliev M.** Echo-based screening of rheumatic heart disease in children: A cost-effectiveness Markov model. Journal of Medical Economics. 2015;18(6):410–9.

30. **Watkins D, Lubinga SJ, Mayosi B, Babigumira JB.** A cost-effectiveness tool to guide the prioritization of interventions for rheumatic fever and rheumatic heart disease control in African nations. PLOS Neglected Tropical Diseases. 2016;10(8):e0004860.

31. **Nordet P, Lopez R, Duenas A,** et al. Prevention and control of rheumatic fever and rheumatic heart disease: The Cuban experience (1986-1996-2002). Cardiovascular Journal of Africa. 2008.

32. **Morgan L.** The financial crisis and global health. Global Health Magazine (http://wwwglobalhealthmagazinecom/top_stories/what_does_the_financial_crisis_mean_for_global_health). 2009;2009:1-.

33. **do Espirito Santo Freire K, de Carvalho MA, Brito TA, Menezes MS.** Valve surgery: Results of the Brazilian public health system. Brazilian Journal of Medicine and Human Health. 2014;2(2).

34. **Ploutz M, Lu JC, Scheel J,** et al. Handheld echocardiographic screening for rheumatic heart disease by non-experts. Heart. 2016;102(1):35–9.

35. **Briggs AH, Claxton K, Sculpher MJ.** Decision modelling for health economic evaluation. Oxford University Press. 2006.

36. **LaMorte W.** Relationship among prevalence, incidence rate, and average duration of disease Boston. Boston University School of Public Health. 2016 [updated June 8, 2016. Available from: http://sphweb.bumc.bu.edu/otlt/MPH-Modules/EP/EP713_DiseaseFrequency/EP713_DiseaseFrequency7.html.

37. **Ferguson N, Datta S, Brock G.** MSSurv, an R Package for nonparametric estimation of multistate models. Journal of Statistical Software. 2012;50(14):1–24.

38. Australia (ARF/RHD writing group) National Heart Foundation of Australia, and the Cardiac Society of Australia and New Zealand. Australian Guideline for
Prevention, Diagnosis and Management of Acute Rheumatic Fever and
Rheumatic Heart Disease. In: of MS, Research H, editors. 2nd edition ed. Darwin, Australia:2012.

39. **Albuquerque DC, Neto JD, Bacal F,** et al. I Brazilian Registry of Heart Failure - Clinical Aspects, Care Quality and Hospitalization Outcomes. Arquivos Brasileiros de Cardiologia. 2015;104(6):433–42.

40. **Pelajo CF, Lopez-Benitez JM, Torres JM, de Oliveira SK.** Adherence to secondary prophylaxis and disease recurrence in 536 Brazilian children with rheumatic fever. Pediatric Rheumatology. 2010;8(1):22.

41. **Mussi CM, Ruschel K, Souza ENd,** et al. Home visit improves knowledge, self-care and adhesion in heart failure: Randomized clinical trial HELEN-I. Revista Latino-Americana de Enfermagem. 2013;21(SPE):20–8.

42. **da Fonseca VBP, De Lorenzo A, Tura BR, Pittella FJM, da Rocha ASC.** Mortality and morbidity of patients on the waiting list for coronary artery bypass graft surgery. Interactive Cardiovascular and Thoracic Surgery. 2018;26(1):34–40.

43. **Ribeiro AHS, Wender OCB, de Almeida AS, Soares LE, Picon PD.** Comparison of clinical outcomes in patients undergoing mitral valve replacement with mechanical or biological substitutes: A 20 years cohort. BMC Cardiovasc Disord. 2014;14:146.

44. **Diamantino A, Beaton A, Aliku T,** et al. A focussed single-view hand-held echocardiography protocol for the detection of rheumatic heart disease - CORRIGENDUM. Cardiology in the Young. 2018;28(4):619.

45. **Neves ACMd, Garcia LP.** Youth mortality in Brazil: Profile and trends in the period 2000–2012. Epidemiologia e Serviços de Saúde. 2015;24(4):595–606.

46. **de Aquino Xavier RM, Azevedo VMP, Godoy PH,** et al. Medium-term outcomes of 78,808 patients after heart valve surgery in a middle-income country: A nationwide population-based study. BMC Cardiovascular Disorders. 2017;17(1):302.
